# Human placenta-derived mesenchymal stem cells stimulate neuronal regeneration by promoting axon growth and restoring neuronal activity

**DOI:** 10.3389/fcell.2023.1328261

**Published:** 2023-12-22

**Authors:** Elvira H. de Laorden, Diana Simón, Santiago Milla, María Portela-Lomba, Marian Mellén, Javier Sierra, Pedro de la Villa, María Teresa Moreno-Flores, Maite Iglesias

**Affiliations:** ^1^ Facultad de C.C. Experimentales, Universidad Francisco de Vitoria, Madrid, Spain; ^2^ Departamento de Biología de Sistemas, Unidad de Fisiología, Facultad de Medicina y Ciencias de la Salud, Universidad de Alcalá, Alcalá de Henares, Spain; ^3^ Departamento de Anatomía, Histología y Neurociencia, Facultad de Medicina, Universidad Autónoma de Madrid, Madrid, Spain

**Keywords:** mesenchymal stem cells, neuroregeneration, neurotrophic factors, hypoxia, electrophysiology

## Abstract

In the last decades, mesenchymal stem cells (MSCs) have become the cornerstone of cellular therapy due to their unique characteristics. Specifically human placenta-derived mesenchymal stem cells (hPMSCs) are highlighted for their unique features, including ease to isolate, non-invasive techniques for large scale cell production, significant immunomodulatory capacity, and a high ability to migrate to injuries. Researchers are exploring innovative techniques to overcome the low regenerative capacity of Central Nervous System (CNS) neurons, with one promising avenue being the development of tailored mesenchymal stem cell therapies capable of promoting neural repair and recovery. In this context, we have evaluated hPMSCs as candidates for CNS lesion regeneration using a skillful co-culture model system. Indeed, we have demonstrated the hPMSCs ability to stimulate damaged rat-retina neurons regeneration by promoting axon growth and restoring neuronal activity both under normoxia and hypoxia conditions. With our model we have obtained neuronal regeneration values of 10%–14% and axonal length per neuron rates of 19-26, μm/neuron. To assess whether the regenerative capabilities of hPMSCs are contact-dependent effects or it is mediated through paracrine mechanisms, we carried out transwell co-culture and conditioned medium experiments confirming the role of secreted factors in axonal regeneration. It was found that hPMSCs produce brain derived, neurotrophic factor (BDNF), nerve-growth factor (NGF) and Neurotrophin-3 (NT-3), involved in the process of neuronal regeneration and restoration of the physiological activity of neurons. In effect, we confirmed the success of our treatment using the patch clamp technique to study ionic currents in individual isolated living cells demonstrating that in our model the regenerated neurons are electrophysiologically active, firing action potentials. The outcomes of our neuronal regeneration studies, combined with the axon-regenerating capabilities exhibited by mesenchymal stem cells derived from the placenta, present a hopeful outlook for the potential therapeutic application of hPMSCs in the treatment of neurological disorders.

## Introduction

Since the classic studies of Ramón y Cajal, the low regenerative capacity of the neurons of the Central Nervous System (CNS) (Ramon y Cajal, 1928) has been known. Since then, various strategies have been used to achieve neuronal regeneration, such as blocking axonal growth inhibitors or with cell therapy, using transplants of different cell types (Horner and Gage, 2000; Jones et al., 2001; Merkler et al., 2001; Raisman, 2001; Bradbury et al., 2002; Moreno-Flores et al., 2002; Okano, 2002; David and Lacroix, 2003; Woolf, 2003; Barnett et al., 2004; Nikulina et al., 2004; Pearse et al., 2004; Sandvig et al., 2004; Silver and Miller, 2004; Sivasankaran et al., 2004; Moreno-Flores and Avila, 2006; Thuret et al., 2006; Gómez et al., 2018). For *in vivo* or future clinical studies, it would be essential to determine *in vitro* the neuroregenerative capacity of the cell populations that would be used in cell therapy. One of the best models to study *in vitro* and quantify cell-induced adult axonal regeneration is the co-culture of adult axotomized retinal ganglion cells (RGCs) with cells putatively capable of inducing axonal regeneration (olfactory ensheathing glia cells—OEGs, Schwann cells, astrocytes, etc.) (Moreno-Flores et al., 2003; García-Escudero et al., 2010; Lim et al., 2010; Portela-Lomba et al., 2020). Our group has carried out studies in co-cultures of RGCs with OEG populations that have made possible to advance in the characterization of the molecular bases of the OEG-dependent regenerative capacity. We have shown that the ability of these cells to induce adult axonal regeneration in co-culture depends on several molecules (Moreno-Flores et al., 2002; Moreno-Flores and Avila, 2006; Gómez et al., 2018): they secrete neurotrophic factors (Woodhall et al., 2001; Pastrana et al., 2007; Runyan and Phelps, 2009), they produce extracellular matrix proteases that contribute to degrading the perineuronal network that stabilizes the environment of adult neurons (Pastrana et al., 2006); produce proteases that stimulate axon regeneration (Simón et al., 2011), etc.

Stem cells are unspecialized cell precursors that are able to self-renew and differentiate into one or more specialized cell types in response to specific signals (Bianco and Robey, 2001; Pardal et al., 2003; Asanuma et al., 2010; Chagastelles et al., 2010). Mesenchymal stem cells (MSCs) are multipotential and orchestrate tissue development, maintenance, and repair (Samsonraj et al., 2017; Shammaa et al., 2020; Zhou et al., 2021; Hoang et al., 2022).

It has been shown, using animal models of spinal cord injury (SCI), that different stem cells, both embryonic and adult, undifferentiated or differentiated in culture with different protocols, are capable of promoting neuro-regeneration after injury, as well as functional recovery (Zipori, 2005; Moreno-Manzano et al., 2009; Erceg et al., 2010).

The regenerative potential of mesenchymal stem cells of different origins has been studied and contrasted by numerous research groups (Li et al., 2022; Lu et al., 2022; Namiot et al., 2022). The source from which organ-specific mesenchymal stem cells have traditionally been extracted is bone marrow. These cells can generate all cell types of the blood and the immune system (McCracken et al., 2016), they can be grown both *in vitro* in the laboratory and *in vivo* (using animal models) and have been tested in tissue repair experiments (Lloyd, 2002; Li et al., 2011). Transplantation of bone marrow-derived cells into SCI models has been reported to promote axonal regeneration, reduce lesion size, and improve functional *outcome* (Chopp et al., 2000; Hofstetter et al., 2002; Ankeny et al., 2004; Kamada et al., 2005; Himes et al., 2006; Shichinohe et al., 2008; Someya et al., 2008; Zurita et al., 2008; Cizkova et al., 2011). The precise cell type within bone marrow responsible for these beneficial effects is not fully established but is thought to reside within the marrow stromal, corresponding to the MSC population (Li et al., 2002; Iihoshi et al., 2004). Additionally, transplantation after SCI of bone marrow MSCs gene-modified to secrete BDNF give way to increased corticospinal neurons survival in primary motor cortex as compared with the unmodified MSCs and promoted effects in locomotor recovery not observed with the control MSCs (Sasaki et al., 2009).

However, the use of bone marrow stem cells has some limitations due to the invasiveness of the extraction method and the decrease in their proliferation and differentiation capacity with the maintenance of cells in culture (Bonab et al., 2006; Chagastelles et al., 2010). Many researchers are working on finding an alternative to these cells that can be used in clinical applications. Placental tissue is a source of cells of great value in regenerative medicine because human placenta-derived mesenchymal stem cells (hPMSCs) are easily isolated and can be expanded in culture using a suitable medium, they have great phenotypic plasticity, and in addition, due to the fact that the placenta is involved in the maintenance of fetal tolerance during pregnancy, they develop immunomodulatory properties of great importance in clinical applications based on cell therapy. These characteristics point to hPMSCs being suitable candidates for use in cell therapies for CNS injury (Samsonraj et al., 2017; Trallori et al., 2019; Andrzejewska et al., 2021; Moonshi et al., 2022).

For all these reasons, in this work we have determined and quantified the ability of hPMSCs to induce adult axonal regeneration by using our model of co-culture with adult axotomized RGCs, under normoxic and hypoxic conditions. It is described that certain features of hPMSCs are stimulated when subjected to low oxygen concentrations in culture, including proliferation, migration, and neuroprotective potential (Mohyeldin et al., 2010; Haque et al., 2013; Youssef et al., 2014; Gu et al., 2016; Zhu et al., 2016; Li et al., 2017; Zhang et al., 2019). Furthermore, we understand that hypoxic conditions are representative of the physiological reality in some time course events after a CNS trauma or some neurological pathologies, such as a ischemic stroke. Therefore, we considered performing experiments simultaneously under normoxia and hypoxia conditions, which would allow us to compare the results in both environments.

Moreover, to assess whether the regenerative capabilities of hPMSCs are entirely contact-dependent effects or they are also mediated through paracrine mechanisms, we carried out transwell co-culture and conditioned medium experiments confirming the role of secreted factors in RGCs’ axonal regeneration.

Finally, through the application of the patch clamp technique, we assessed the functional recovery of regenerated neurons, thus confirming their electrophysiological activity. This provides valuable insights into the realm of neural regeneration and its potential implications for treating neurological disorders.

## Materials and methods

### Culture of hPMSCs

hPMSCs (Cellular Engineering Technologies, Coralville, United States, cat# HMSC.AM-100) were grown in HGCM composed of high glucose Dulbecco’s modified Eagle’s medium (DMEM) (4.5 g/L) (Gibco, Grand Island, New York, United States, cat# 11504496) supplemented with 10% fetal bovine serum (FBS) (Gibco, reference 10270106), an antibiotic and antifungal solution (Pen/Strep/Fung 10k/10k/25 μg, Lonza, Basel, Switzerland, cat# H317-745E), 2 mM L-glutamine (Lonza, cat# H3BE17-605E), and basic fetal growth factor (hFGF basic) (Gibco, cat# PHG0026). Cell cultures were incubated at 37°C under normoxic conditions (5% CO_2_ and 20% O_2_) and hypoxic conditions (5% CO_2_ and 1% O_2_). The maintenance of hypoxia conditions in the cultures was monitored by assays by immunodetection of hypoxia inducible factor (HIF).

### Olfactory ensheathing cells (OECs)

OEGs line TS12 (Plaza et al., 2016) was maintained in ME medium, composed of DMEM/F12 (Gibco, cat# 11320074) supplemented with 10% FBS, 2 mM glutamine (Lonza), 20 μg/mL pituitary extract (Gibco, cat# 13028014), 2 µM forskolin (Sigma, cat# F6886) and an antibiotic and antifungal solution (Pen/Strep/Fung 10k/10k/25 μg, Lonza).

### Proliferation capacity of hPMSCs

To examine the proliferation capacity of the hPMSCs *in vitro*, the cumulative population doubling (PD) was calculated over 30 days (from passage 3–8). The hPMSCs were placed in triplicate into 10 cm^2^ multiwell dishes at a concentration of 10^4^ cells/cm^2^ and subcultured after 5 days at the same density. The cells were counted using a hemocytometer. The cumulative cell doubling of the cell populations was plotted against time in the culture to determine the growth kinetics of hPMSCs expansion. The number of population doubling was determined by counting the number of adherent cells at the start and end of each passage. The population doubling was calculated at every passage according to the equation: log_2_ (number of harvested cells/number of seeded cells). The finite population doubling was determined by the cumulative addition of the total numbers generated from each passage until the cells stopped dividing.

### Mesodermal differentiation of hPMSCs

For adipogenic differentiation hPMSCs were seeded at an inoculation density of 2.4 × 10^5^ cells/well in 6-well plates until they reached 100% confluence and were then induced by three cycles of induction/maintenance with Differentiation media BulletKits—adipogenic (Lonza, cat# PT-3004) according to the manufacturer’s instructions. Adipogenesis was assayed after 18 days of culture by staining of intracellular lipid droplets with Oil Red O (Sigma, St. Louis, Missouri, United States. cat# O0625. Detailed protocol in Supplementary Document). The monolayer cultures were incubated with Oil Red O solution for 10 min at ambient temperature and examined by microscopy.

For osteogenic differentiation hPMSCs were seeded at an inoculation density of 3 × 10^4^ cells/well in 6-well plates until they reached 60%–80% confluence and cultured in osteogenic induction medium Differentiation media BulletKit™—osteogenic (Lonza, cat# PT-3002) according to the manufacturer’s instructions. After 21 days in osteogenic induction medium, mineral deposits were observed by Alizarin Red (Sigma, cat# A5533) staining. For Alizarin Red staining, the cells were fixed with 10% formalin for 5 min. The formalin was removed, and cells were washed twice with water. Then, the cells were incubated with Alizarin Red S staining solution (Detailed protocol in Supplementary Document) for 20 min at ambient temperature and examined by microscopy.

For chondrogenic differentiation micromass cultures were generated by seeding 5 µL droplets of 1.6 × 10^7^ cells/mL cell solution in the center of multi-well plate wells. After cultivating micromass cultures for 2 h StemPro^®^ Chrondrogenesis differentiation medium (Gibco, cat# A10071-01) was added. After 14 days of incubation, the micromasses were rinsed with PBS and fixed with 4% paraformaldehyde solution for 30 min. Chondrogenic differentiation was observed by Alcian Blue staining (Merck KGaA, Darmstadt, Germany. cat# A3157. Detailed protocol in Supplementary Document) and examined by microscopy.

### Flow cytometry analysis

To confirm MSCs phenotype of cells grown in normoxic and hypoxic conditions were analyzed by flow cytometry. Antibodies against the following human antigens were used: CD105-FITC (Miltenyi Biotect, Bergisch Gladbach, Germany, cat# 130-112-327, 1:50), CD90-FITC (Miltenyi Biotec, cat# 130-114-901, 1:50), CD44-VioBlue (Miltenyi Biotec, cat# 130-113-906, 1:50), CD73-APC (Miltenyi Biotec, cat# 130-111-909, 1:50), MSC Phenotyping Cocktail-PE (CD34, CD14, CD19, CD45, Miltenyi Biotec cat# 130-125-285, dilution according to the manufacturer’s instructions). Briefly, cells were seeded at a concentration of 10^4^ cells/cm^2^ and maintained in culture until they reached 80%–90% confluence. Then cells were trypsinized, surface labeled, washed and then analyzed using a flow cytometer (Miltenyi Biotech). The data were analyzed with MACSQuantify™ Software.

### Western blot assays

Protein lysates at different passages (from 3 to 5) and different oxygen concentration cultures of hPMSCs were obtained using lysis buffer (NaCl 150 mM, IGEPAL^®^ 1%, DOC 0.5%, SDS 0.1%, Tris pH 8 50 mM) containing protease inhibitors (×50, Roche, Mannheim, Germany). Protein concentrations were determined using bicinchoninic acid assay (Thermo Fisher Scientific, Waltham, MA, United States, cat# A55861). Equal amounts of protein (30 µg) were separated by 10% sodium dodecyl sulfate-polyacrylamide gel electrophoresis and transferred to polyvinylidene difluoride membranes (Roche). Membranes were blocked and incubated with primary antibodies at 4°C overnight: anti-BDNF (1:1,000, rabbit, Abcam, cat# ab108319), anti-NGF (1:1,000, rabbit, Abcam, cat# ab52918), anti-NT-3 (1:1,000, rabbit, Abcam, cat# ab75960), anti-HIF (1:1,000, rabbit, Abcam, cat# ab51608) or anti-β-actin (1:1,000, mouse, Merck, cat# A5441). Next day, membranes were incubated with horseradish peroxidase-conjugated goat anti-rabbit (1:10,000, Cell Signaling Technology, cat# 7470) or sheep anti-mouse (1:10,000, Merck, cat# AC111P) secondary antibodies for 1 h at ambient temperature. After applying ECL substrate (Amersham, Buckinghamshire, United Kingdom, cat#RPN2209) the protein bands were detected by chemoluminiscence in ChemiDoc MP Imaging System (Bio-Rad) and the relative protein expression to β-actin was analyzed by ImageJ software.

### Axonal regeneration assays by direct co-culture

Axonal regeneration assays were performed and analyzed as previously described. Retinal tissue was extracted from 2-month-old rats, and then digested with papain using Worthington’s Papain System (20 units/mL; Worthington-Biochemical Corporation, Lakewood, NJ, cat# LK003150). Axotomized adult retinal neurons were plated onto hPMSCs (5 × 10^4^, 8 × 10^4^ and 10^5^) and OEG (1.1 × 10^5^) monolayers and the cultures were maintained in serum-free neurobasal medium (Gibco, cat# 21103049), supplemented with B-27 (Gibco, cat# 17504044) (NB-B27) for 96 h in normoxia and hypoxia. Axotomized adult retinal neurons were also plated onto 10 µg/mL poly-L-lysine (PLL) (Sigma, cat# P6282) coated dishes as negative control. Co-cultures were fixed with 4% paraformaldehyde (PFA).

### Axonal regeneration assays by indirect co-culture

Axotomized retinal neurons were plated onto 10 µg/mL PLL (Sigma) coated dishes and cultured in NB-B27 medium. hPMSCs were seeded directly onto 24-well 0.4 µm transwell inserts at a density of 10^4^ cells/cm^2^, cultured in NB-B27 medium and maintained in normoxia and hypoxia for 96 h before fixing them with 4% PFA.

### Axonal regeneration assays using conditioned media

hPMSCs were seeded at a density of 10^4^ cells/cm^2^ in HGCM. When cultures reached approximately 90% confluence, hPMSCs were rinsed with PBS (PBS EDTA pH 7.5; Lonza, cat# 15374875) and maintained in NB-B27 medium for 96 h. Conditioned medium was collected and centrifuged at 1,200 g for 5 min.

Axotomized neurons were plated as described above. Conditioned medium or equivalent volume of NB-B27 medium (control) was added to the neurons. The cells were incubated for 96 h and fixed with 4% PFA.

Axonal regeneration events were thoroughly characterized and quantified as described previously (Moreno-Flores et al., 2003; Pastrana et al., 2006; Portela-Lomba et al., 2020). Co-cultures were analyzed by immunocytochemistry employing an antibody against a phosphorylated form of MAP1B and Neurofilament-H (NF-H) axonal proteins (SMI31; Sternberger Monoclonal Inc., Baltimore, MD; 1:500, cat#801601) and an antibody, 514, which recognizes high molecular weight microtubule-associated protein 2 (MAP2A,B; 1:400) (Sánchez Martin et al., 2000). The percentage of retinal neurons with an axon was determined by counting the number of MAP2A,B positive neurons that bear a polarized neurite that can be labelled with the antibody against the phosphorylated forms of MAP1B and NF-H proteins. The samples were observed in an inverted fluorescent microscope with a ×40 immersion objective (DMi8, Leica, Wetzlar, Germany) and a minimum of 20 fields (containing a minimum of 200 neurons) were taken randomly.

Two parameters were determined: percentage of neurons that have regenerated axons (number of neurons with an axon versus total number of counted neurons) and the mean axonal length of the regenerated axons (µm)/neuron (ratio between the total length of the regenerated axons and the total number of counted neurons). Fluorescence signals were analyzed with ImageJ software (ImageJ, NIH) and the length of the axons was determined using the Neuron J plugin.

### Immunocytochemistry

Direct and indirect co-cultures, and cultures with conditioned media, were fixed with 4% PFA to perform immunocytochemistry analysis. Samples were permeabilized and blocked in blocking solution (×1 PBS, 0.1% triton, 1% FBS) for 30 min at room temperature. Next, they were incubated with the corresponding primary antibody in blocking solution at the optimal dilution for each of them. The primary antibodies used were SMI31 and 514. Incubation was carried out at 4°C overnight. After washing, the cells were incubated for another hour in a solution of PBS-TS containing the appropriate fluorescent secondary antibody, at its optimal dilution. Fluorescence-conjugated secondary antibodies used were Alexa Fluor 488 and Alexa Fluor 594, respectively.

Finally, the samples were washed with PBS and mounted with Fluoromount (Southern Biotech, Birmingham, Alabama, United States, cat#0100-01). The labelled preparations were visualized using an inverted fluorescent microscope with a ×40 immersion objective (DMi8, Leica).

### Study of properties of regenerated axons

To detect the expression of mature synaptic vesicles and voltage-gated sodium channels (VGSCs), direct and indirect co-cultures were fixed, permeabilized and blocked as previously described. MAP2A,B (514) (Simón et al., 2011), SV2A (kind gift from Dr. Morcillo, University of Extremadura, Spain) and Nav1.1 α subunit (SCN1A) (Sigma, cat# S8809) primary antibodies were incubated at 4°C overnight. Fluorescence-conjugated secondary antibodies used were Alexa Fluor 488 and Alexa Fluor 594.

### Brain derived neurotrophic factor (BDNF) and nerve growth factor (NGF) quantification

BDNF and NGF levels in direct and indirect co-cultures, and in conditioned media, were evaluated using Human BDNF ELISA kit (Abcam, cat# ab1212166) and Human beta Nerve Growth Factor ELISA Kit (Abcam, cat# ab193760) following the manufacturer’s instructions.

### Electrophysiology

Electrophysiological recording of cell voltage and ionic currents were performed under the whole cell configuration of the patch clamp technique (Hamill, Marty, Neher, Sakmann and Sigworth, 1981) using an Axopatch 200A amplifier (Axoninstruments, FosterCity, CA, United States). Borosilicate pipettes (1.2 mm outer diameter) with a tungsten internal filament, were made using a vertical pipette puller (Narishige mod. P88, Narishige, Tokyo, Japan). The inner diameter of the pipette after pulling was approximately 0.5–1 μm. Filling of the pipette was carried out with an intracellular solution containing (in mM): 10 NaCl, 110 KCl, 5 EGTA, 0.5 CaCl_2_, 1 MgCl_2_, and 10 glucose (pH 7.4). The electrical resistance of the pipettes was measured, in the range of 8–12 MΩ. The seal resistance was approximately 1–3 GΩ. Liquid junction potential was routinely corrected. The Ag-AgCl indifferent electrode was connected via an agarose bridge to the perfusion. The cell voltage was maintained at −80 mV and depolarizing pulses of 30 ms duration were applied in steps of 5 mV. Both the holding voltage and the pulses were generated using a personal computer connected to the CED plus (Cambridge Electronic Design Ltd., Cambridge, England). Data were sampled at 0.2–10 kHz after low-pass filtering with an appropriate cut-off for each sampling frequency. Data analysis was performed offline using a personal computer.

### Statistical analysis

Data are presented as the mean ± standard deviation (SD). Multiple comparisons, one-way ANOVA followed by Dunnet´s *post hoc* test was used to evaluate the differences in axonal regeneration. An alpha value of *p* ≤ 0.05 was used for statistical significance. Multiple comparisons, two-way ANOVA followed by Sidak´s *post hoc* test was used to evaluate the differences in proliferation capacity of hPMSCs and pro-neurotrophic factor expression. An alpha value of *p* ≤ 0.05 was used for statistical significance.

## Results

### Culture and characterization of hPMSCs

Prior to evaluate neural regeneration potential of hPMSCs, we confirmed that the cells were in agreement with the criteria of International Society for Cellular Therapy published in 2006 (Dominici et al., 2006).

**FIGURE 1:**
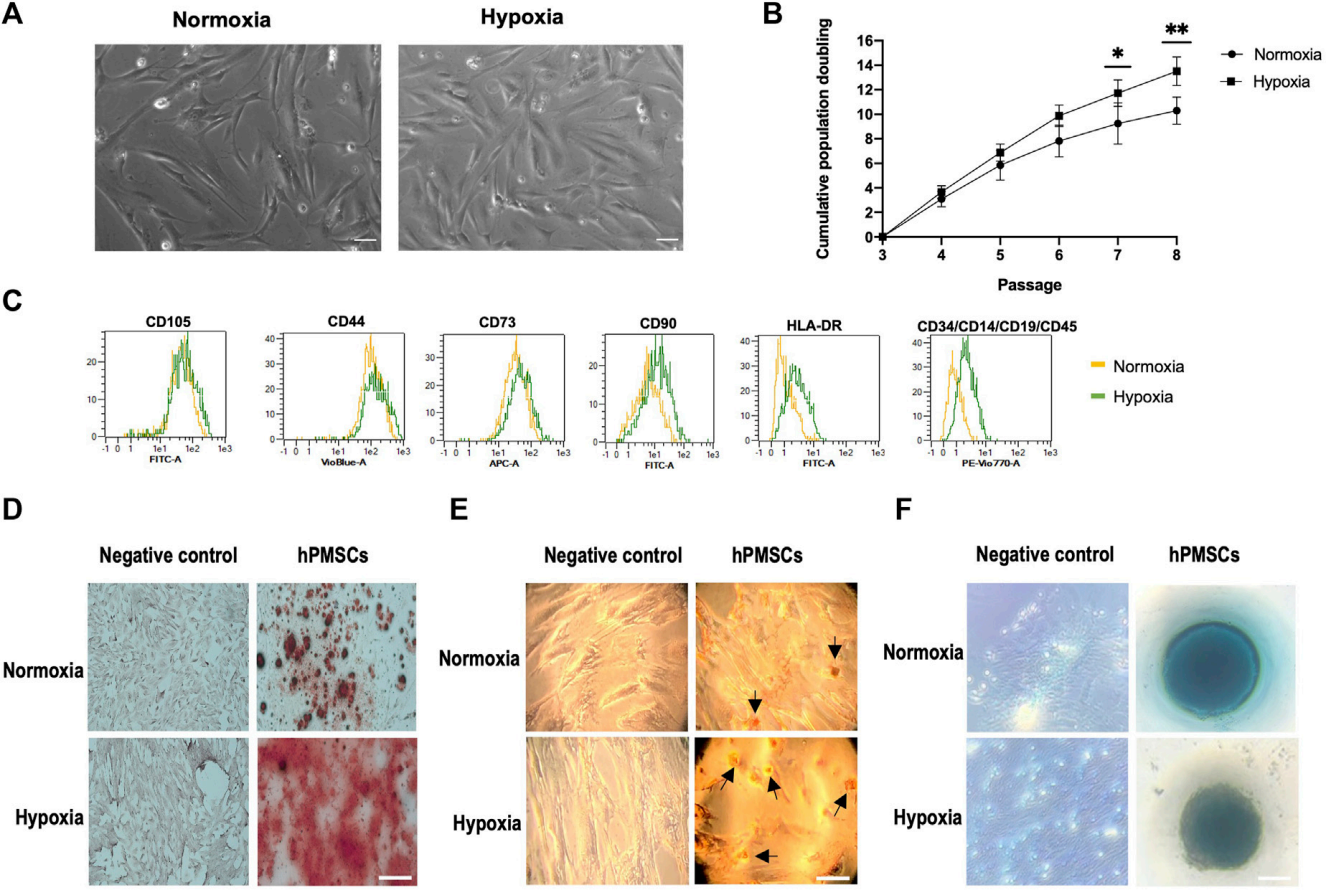
Characterization of hPMSCs. **(A)** Morphology at passage 3. Both normoxic and hypoxic cultures show highly homogeneous spindle-like shape. Scale bar: 500 µm **(B)** Proliferation capacity. Cumulative population doublings of hPMSCs grown under hypoxia (square) are higher than cultures grown in normoxia (dot). **(C)** Flow cytometry shows specific hMSCs marker expression pattern: positive for CD105, CD44, CD90, CD73 and negative for CD14, CD19, CD34, CD45 and HLA-DR. **(D)** Osteogenic differentiation was confirmed by Alizarin red staining of calcium deposits. **(E)** Adipogenesis differentiation was followed by Oil Red O staining of lipids vacuoles (black arrows indicate lipid droplets). **(F)** Alcian Blue staining of proteoglycans demonstrated chondrogenesis differentiation. Scale bar: 100 μm. Two-way ANOVA and *post hoc* Sidak test for multiple comparisons between means (**p ≤ 0.005; *p ≤ 0.05).

Mesenchymal cells attached to cell culture plastic flasks and showed a typical spindle-type shape morphology when observed by inverted phase contrast microscopy (Figure 1A). No differences in morphology between oxygen conditions were detected.

The comparative growth kinetics of the hPMSCs grown in normoxia and hypoxia from passages 3 to 8 revealed that hypoxic cultures had a higher growth potential, measured as cumulative PDs (Figure 1B). In fact, when comparing normoxic and hypoxic cultures the difference in PDs at passage 7 and 8 is statistically significant.

To assess the expression of mesenchymal and hematopoietic markers an immunophenotypic analysis was performed. Flow cytometry results indicated that hPMSCs expressed typical immunophenotypic characteristics consistent with those of a mesenchymal lineage. Cells cultured in both oxygen conditions were positive for surface markers CD105, CD44, CD90 and CD73 and negative for CD34, CD14, CD45, CD19, and HLA-DR (Figure 1C).

We investigated the differentiation potential of hPMSCs by inducing them into osteoblasts, adipocytes and chondrocytes *in vitro*. Alizarin red staining revealed significant calcium deposition in treated cells, thus confirming osteogenesis (Figure 1D), Oil Red O-stained lipid drops were observed in differentiated hPMSCs indicating adipogenesis (Figure 1E), and the presence of proteoglycan staining with Alcian blue proved chondrogenesis (Figure 1F). Interestingly, hPMSCs grown under hypoxic conditions showed a substantial increase in calcium deposition when compared to hPMSCs grown in normoxia. Similarly, hypoxic hPMSCs cultures showed a higher number of lipids droplets. These results suggest that hypoxia promotes hPMSCs differentiation potential.

Taken together, these results confirm that the cells of this study were mesenchymal stem cells. In addition, these data reveal that low oxygen concentration has no significant *in vitro* effect on the morphology and phenotype profiles of hPMSCs but it can promote both hPMSCs proliferation and differentiation abilities.

### Neuroregenerative effect of hPMSCs

To evaluate hPMSCs neuroregenerative potential an *in vitro* regeneration assay was performed. All analyses were conducted on cells at passages 2–5, simultaneously cultured under normoxic and hypoxic conditions.

**FIGURE 2:**
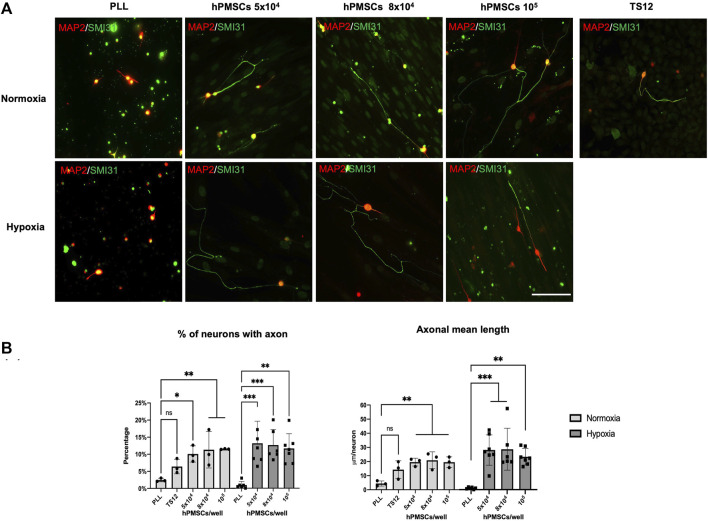
Neuroregenerative effect of hPMSCs. Axotomized adult retinal neurons were co-cultured with hPMSCs at different densities. TS12 cells were used as low regenerative control. Axotomized neurons were also plated onto poly-L-lysine substrate (PLL) to measure the intrinsic neuroregenerative capacity. **(A)** After 96 h of culture, cells were fixed and immunostained with antibodies SMI-31 (MAP1B/NF-H axonal marker, green) and 514 (MAP2A,B somatodendritic marker, red). Representative images are shown. Scale bar: 100 μm. **(B)** Axonal regeneration quantification: percentage of retinal neurons with an axon and axon mean length (μm/neuron). ANOVA and *post hoc* Dunnet’s test. *p < 0.05, **p < 0.005 and ***p < 0.001. (*n* = 3 normoxia; *n* = 6 hypoxia).

Different densities of hPMSCs (5 × 10^4^, 8 × 10^4^ and 10^5^) were co-cultured with axotomized 2-month-old rat retinal ganglion cells (RGCs) in both normoxia and hypoxia, for 96 h. In parallel, an immortalized human olfactory ensheathing cell line (TS12) was used as a low regenerative capacity control (Plaza et al., 2016). Wells treated with the substrate PLL with no cells attached were used as a negative control for RGCs axonal regeneration capacity. Neuroregeneration was detected by immunofluorescence using SMI31 antibody (against MAP1B and-NF-H) as an axonal marker and 514 antibody (against MAP2A,B) to observe the somatodendritic compartment (Figure 2A). Two parameters were then quantified: the percentage of neurons with an axon, and the mean axonal length per neuron (µm/neuron).

Axotomized RGCs showed a low regeneration capacity in normoxic PLL single cultures (2.5% ± 0.5 GCs with an axon). This capacity was lower in hypoxic atmosphere (1.04% ± 1.1 GCs with an axon) (Figure 2B).

However, the presence of hPMSCs significantly increased RGCs axonal regenerative capacity whereas TS12 cells did not (Figure 2B). The increment of RGCs with regenerated axon in the presence of hPMSCs was similar in both oxygen conditions, being slightly more efficient in hypoxia (14.58% ± 7.8, 14.01% ± 5.0, 12.90% ± 4.7 RGCs with an axon when co-cultured with 5 × 10^4^, 8 × 10^4^ and 10^5^ hPMSCs respectively) than in normoxia (10.08% ± 2.3, 11.33% ± 5.3, 11.52% ± 0.1 RGCs with an axon). Accordingly, mean axonal length in normoxia was 19.57 ± 2.74, 20.88 ± 5.96, 19.52 ± 3.75 mm/neuron when RGCs were seeded with 5 × 10^4^, 8 × 10^4^ and 10^5^ hPMSCs respectively and 26.96 ± 11.18, 25.80 ± 14.28, 23.51 ± 5.63 mm/neuron in hypoxia (Figure 2B). The differences between the percentage of neurons with an axon and axonal mean length/neuron between the different cell densities are not significant. However, the axonal regeneration observed in hypoxia is significantly higher for both quantified parameters than its negative control (PLL), compared to that achieved under normoxia conditions.

Notably, these data demonstrate a high increment in axotomized RGCs regenerative capacity when cultured in the presence of hPMSCs.

### Neurotrophic factors expression by hPMSCs

**FIGURE 3:**
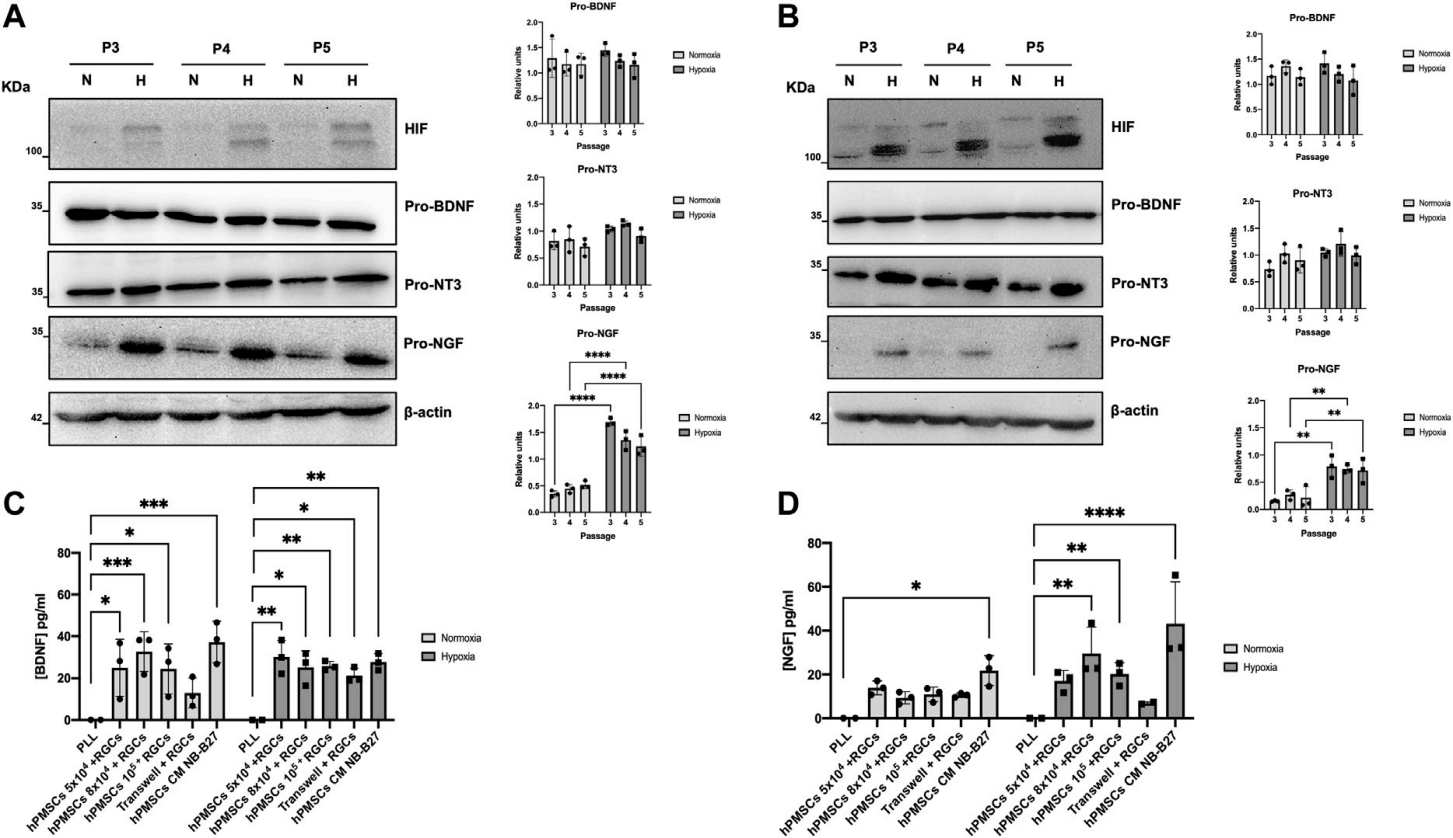
Neurotrophic factors expressed by hPMSCs. Western blotting was performed to analyze the synthesis of pro-BDNF (32 KDa), pro-NT3 (35 KDa), pro-NGF (32 KDa) in hPMSCs cultures at passage 3 (P3), passage 4 (P4), passage 5 (P5), grown in normoxia (N) and hypoxia (H). hPMSCs were cultured in **(A)** DMEM high glucose (4,5 gr/L) or **(B)** NB-B27 media. Expression of the proteins was quantified as arbitrary units using ImageJ software. Values are expressed as the ratio between the corresponding pro-factor and β-actin as a loading control (on the right). Two-way ANOVA and *post hoc* Sidak’s test statistical analysis was performed (****p ≤ 0.0001; *p ≤ 0.05). Maintenance of hypoxic conditions was monitored by immunodetection of hypoxia inducible factor (HIF). Secreted BDNF **(C)** and NGF **(D)** were measured using ELISA-based assays. ANOVA and *post hoc* Dunnet’s test. *p < 0.05, **p < 0.005, ***p < 0.001 and ****p < 0.0001.

Western blot experiments confirmed pro-BDNF, pro-NT3 and pro-NGF synthesis by hPMSCs when grown both in HGCM or NB-B27 media (Figures 3A, B) at any time point studied. After quantification by ImageJ we observed that pro-BDNF synthesis does not depend on culture conditions. The expression of pro-NT3 is similar when hPMSCs are grown in normoxia compared to hypoxia. Finally, we concluded that pro-NGF synthesis by hPMSCs was 2–3 times higher when cells are grown in hypoxia relative to normoxia, in both types of culture media. Cell growth in hypoxia triggers the expression of HIF as it is described (Tirpe et al., 2019).

We confirmed the secretion of neurotrophic factors by hPMSCs. The concentration of secreted BDNF by hPMSCs (5 × 10^4^, 8 × 10^4^ and 10^5^ cells/plate) when maintained in co-culture with RGCs in normoxia is 25 ± 13.68, 32.74 ± 9.49 and 24.4 ± 11.99 pg/mL, respectively. In hypoxia, secreted BDNF achieves a concentration of 30.12 ± 8.04, 25.24 ± 7.92 and 25.83 ± 2.18 pg/mL. The concentrations are lower in indirect Transwell co-cultures (12.97 ± 7.09 pg/mL in normoxia, 21.19 ± 3.61 pg/mL in hypoxia). hPMSCs grown in NB-B27 medium (hPMSCs CM NB-B27) in absence of RGCs secreted a higher concentration of BDNF as a mean, but the difference was not significant attending culture conditions (Figure 3C).

Secreted NGF concentration is slightly higher in hypoxia (17.06 ± 4.76, 29.66 ± 11.93 and 20.23 ± 5.15 pg/mL in 5 × 10^4^, 8 × 10^4^ and 10^5^ hPMSCs + RGCs co-cultures, respectively) than in normoxia (13.93 ± 3.17, 9.33 ± 2.75 and 10.92 ± 3.30 pg/mL) (Figure 3D).

### Neuroregenerative effect of hPMSCs by indirect co-culture and conditioned medium collected from hPMSCs

Our previous results show that hPMSCs provided a permissive substrate that allowed axon regeneration and elongation in axotomized RGCs linked to neurotrophic pro-factors synthesized by hPMSCs. In order to evaluate a possible paracrine effect of neurotrophic factors secreted by hPMSCs two kind of *in vitro* indirect co-culture assays were performed.

**FIGURE 4:**
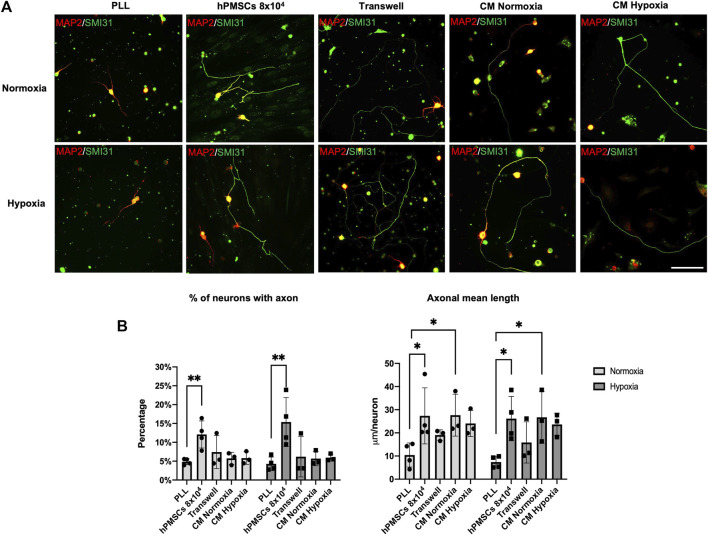
Effect of neurotrophic factors secreted by hPMSCs. Axotomized adult retinal neurons were plated onto poly-L-lysine substrate (PLL) to measure the basal RGCs neuroregenerative capacity. Co-cultures with hPMSCs (8 × 10^4^ cells) were used as positive control. Indirect co-cultures of axotomized neurons and hPMSCs were performed using Transwell inserts. Conditioned media both in normoxic (CM Normoxia) and hypoxic (CM Hypoxia) atmosphere were added to axotomized retinal neuron in culture. **(A)** After 96 h of culture, cells were fixed and immunostained with antibodies SMI-31 (MAP1B/NF-H axonal marker, green) and 514 (MAP2A,B somatodendritic marker, red). Representative images are shown Scale bar: 100µm. **(B)** Axonal regeneration quantification: percentage of retinal neurons with an axon and mean axonal length per neuron (μm/neuron). ANOVA and *post hoc* Dunnet’s test. *p < 0.05 and **p < 0.005. (*n* = 3 normoxia; *n* = 3 hypoxia).

For indirect co-culture assay: axotomized RGCs were plated onto PLL coated dishes. hPMSCs cell suspension (10^4^ cells/cm^2^) in NB-B27 medium was seeded onto the 0.4 µm Transwell inserts. These indirect co-cultures were performed in both normoxia and hypoxia. For secretome activity assay: NB-B27 medium was conditioned by hPMSCs in normoxia or hypoxia and later added to axotomized RGCs cultures on PLL (Figure 4A). Axonal regeneration was characterized and quantified as described previously (Figure 4B).

Results suggest an active role of hPMSCs secreted factors on RGCs neuroregeneration. In indirect Transwell co-cultures, the percentage of RGCs with a regenerated axon is very similar to control cultures (PLL) in normoxia (7.41% ± 4.3) and hypoxia (6.19% ± 5.3). However, mean axonal length per neuron achieved is more than 2 times higher in both conditions (19.07 ± 2.24 mm/neuron in normoxia; 15.87 ± 8.9 mm/neuron in hypoxia).

Similar results were obtained when axotomized RGCs were cultured with hPMSCs CM. Under those conditions, although the percentage of RGC with a regenerated axon is similar to the control (PLL), the mean axonal length per neuron is higher in both normoxia and hypoxia. No differences were observed in the effect on regeneration when CM were conditioned in either normoxic or hypoxic conditions (Figure 4B).

These results strongly suggest that hPMSCs function as a biological substrate that increase the capacity of RGCs to regenerate their axons, partly through extracellular molecules.

### Study of functional properties of regenerated axons

After neuroregeneration potential of hPMSCs was confirmed, we further analyzed the developmental stage of the regenerated axons by the detection of mature synaptic vesicles and voltage-gated sodium channels (VGSCs). Additionally, we explore the axonal complete functionality by measuring their electrophysiological capacity.

**FIGURE 5:**
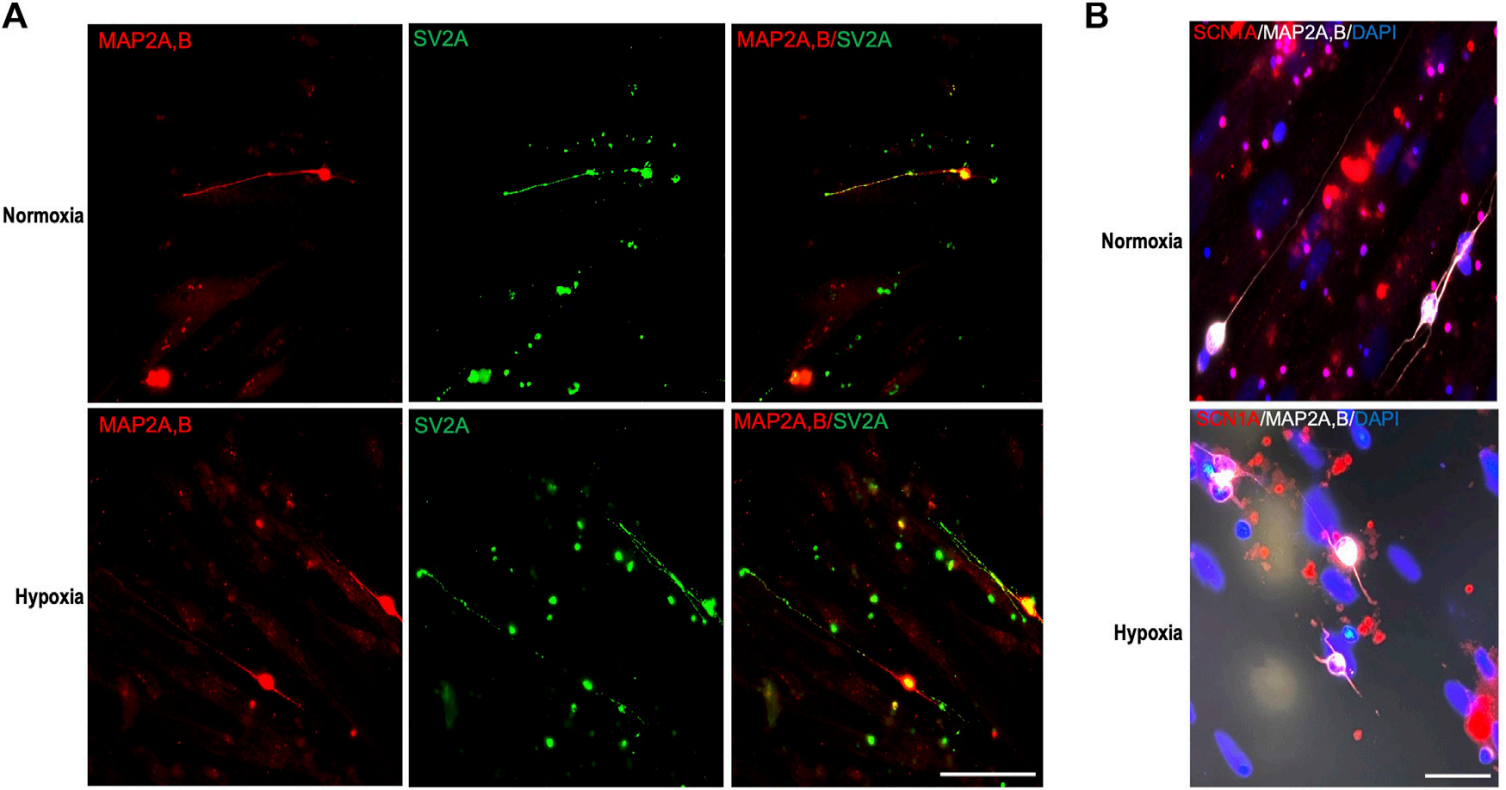
Study of properties of regenerated axons. Axotomized adult retinal neurons were co-cultured with 8 × 10^4^ hPMSCs/well. **(A)** After 96 h of culture cells were fixed and immunostained with antibodies 514, against MAP2A,B (somatodendritic marker, red) and anti-SV2A, against synaptic vesicle membrane glycoprotein 2 (green). Representative images are shown. **(B)** Representative immunofluorescence of voltage-gated sodium channel Nav1.1 α subunit (SCN1A, red), 514 against MAP2A,B (white) and nuclear staining with DAPI (blue). Scale bar panel **(A)** 100 μm; scale bar panel **(B)** 50 μm.

SV2A is a synaptic vesicle membrane glycoprotein expressed exclusively in neurons and endocrine cells essential in neurotransmitter release (Ciruelas et al., 2019). To test the production of mature synaptic vesicles, SV2A protein expression was detected in regenerated axons of RGCs after hPMSCs co-culture. We observed several SV2A positive *puncti* throughout RGCs regenerated axons, with similar size and shape as previously described (Nowack et al., 2010) (Figure 5A). Those images demonstrated the presence of mature synaptic vesicles along those axons, suggesting their ability to release neurotransmitters in both oxygen conditions.

VGSCs are responsible of the initiation and propagation of potentials in excitable cells. VGSCs Nav1.1 α subunit (SCN1A) is expressed predominantly in cell bodies and dendrites and participate in generation of both somatodendritic and axonal action potentials (Rhodes et al., 2004; Catterall et al., 2010) Immunostaining of SCN1A in hPMSCs-induced regenerated RGCs axons revealed in both conditions the presence of VGSCs in RGCs bodies, dendrites, and initial part of the axons (co-stained with MAP2A,B) suggesting their ability to generate action potentials (Figure 5B).

Taken together, these results confirm that RGCs axons, regenerated by hPMSCs induction, may have developed the subcellular and molecular capacity to be fully functional.

Once the neuroregenerative potential of hPMSCs and the expression of VGSC were confirmed, we proceeded to functionally explore the electrical activity of the regenerated neurons. For this purpose, we recorded the ionic currents generated by the cells maintained in normoxic and hypoxic conditions, by using the patch clamp technique, in “whole cell” configuration.

**FIGURE 6:**
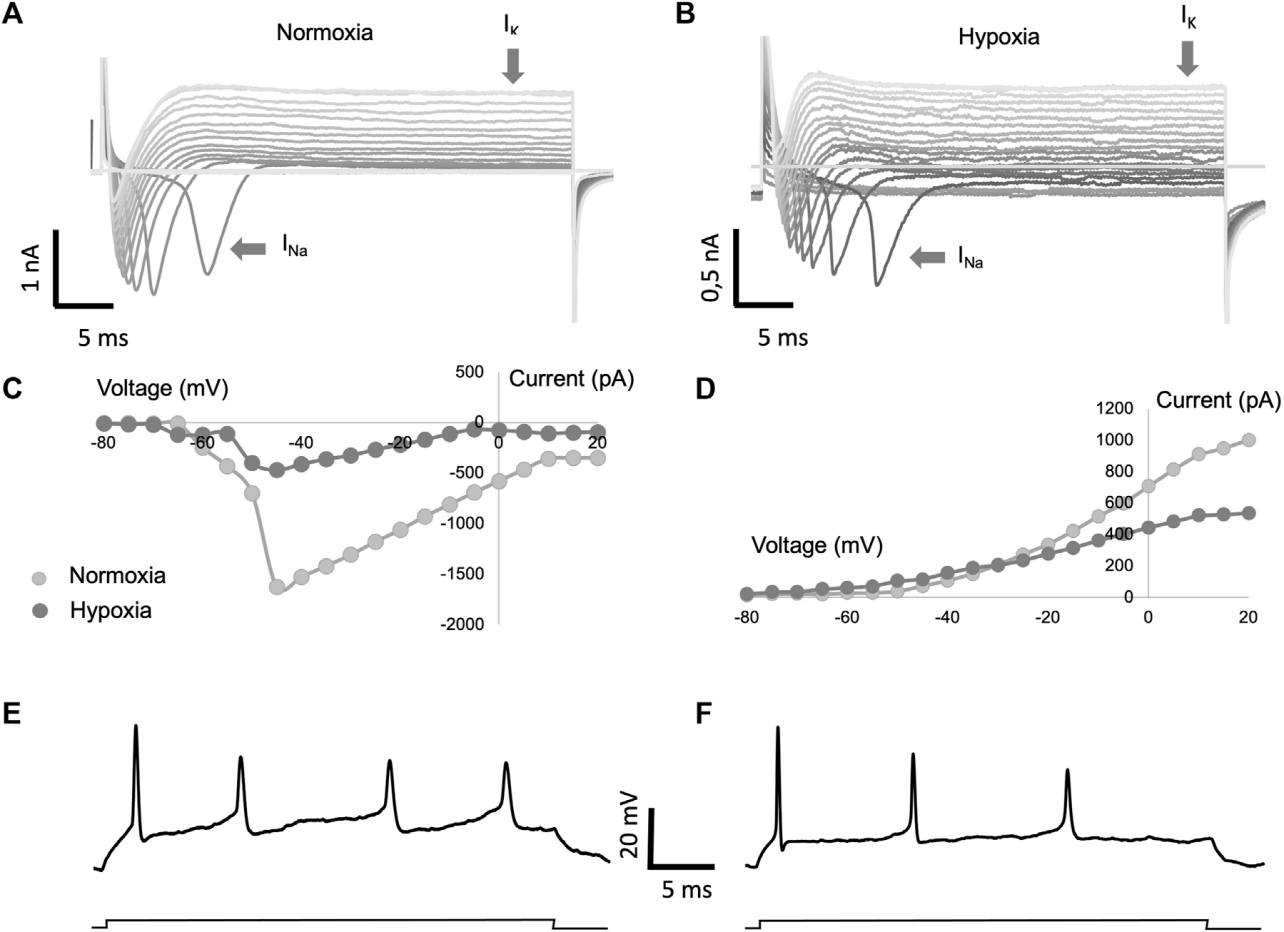
Study of electrical properties of regenerated axons. Axotomized adult retinal neurons were co-cultured with 8 × 10^4^ hPMSCs/well and recorded under voltage/current clamp conditions. **(A,B)** Representative whole cell current recordings from cultured cells in response to voltage pulses of 5 mV increasing step from a holding voltage of −80 mV in conditions of normoxia **(A)** and hypoxia **(B)**. **(C,D)** Current voltage relationship of the sodium currents **(C)** and potassium currents **(D)** averaged from a total of 8 cells in normoxic conditions and 7 cells in hypoxic conditions. **(E,F)** Representative whole cell voltage recordings from cells shown in **(A,B)** in response to a current pulse of 0.1 nA.

In a series of voltage clamping experiments carried out in regenerated cells, the ionic currents activated by cell depolarization were recorded. Depolarizing pulses were applied from a membrane potential of −80 mV in 5 mV step. Figure 6 shows the depolarization-activated currents recorded in a normoxic condition sample (Figure 6A), as well as in a hypoxic condition sample (Figure 6B). In both conditions, it is possible to observe the transient sodium current (I_Na_), followed by the sustained potassium current (I_K_). These experiments, carried out in a group of regenerated axons in normoxia (*n* = 8) and regenerated axons in hypoxia (*n* = 7), allowed us to observe that the amplitude of the sodium currents recorded from regenerated cells in hypoxia was significantly lower than the current from the normoxic regenerated cells (Figure 6C). Similarly, the amplitude of potassium currents showed less amplitude in the hypoxic group than in the normoxic group (Figure 6D). Despite the observed differences, both cell types were able to generate action potentials when recorded in current clamp mode (Figures 6E, F). Therefore, our functional experiments demonstrate that the regenerated cells are active from an electrophysiological point of view.

## Discussion

The evolution of regenerative medicine emphasizes the significance of stem cell-based approaches in therapy development. Among sought-after cell sources, MSCs stand out for their unique qualities, playing a pivotal role in tissue maintenance, repair, and development. MSCs, known for their role in tissue growth and repair, hold promise in regenerative therapies targeting degenerative diseases and various clinical conditions (Bravery et al., 2013; Paradisi et al., 2014; Campbell et al., 2015; Morizane, 2019; Pisciotta et al., 2020; Andrzejewska et al., 2021).

Placenta-derived mesenchymal cells (hPMSCs) are a notable MSC source. They offer accessibility without invasiveness, possess strong immunomodulatory properties, high proliferative and differentiation potential, have the ability to migrate to injury sites *in vivo* and are ethically sound and versatile. For all these reasons, hPMSCs hold promise for regenerative medicine and better patient outcomes (Parolini et al., 2008; Vellasamy et al., 2012; Belmar-Lopez et al., 2013; Kong et al., 2018; Moonshi et al., 2022).

Recognizing the significant potential of these cells as therapy, our research group has directed its efforts towards further exploration and enhanced understanding of hPMSCs’ potential as cellular strategy to achieve neuronal regeneration.

As a preliminary and crucial step in the study we ensured that the properties of the cells in culture remained consistent across passages. Ensuring that cells maintain their intended characteristics over time and culture conditions is crucial for the reliability and reproducibility of experiments and future therapeutic interventions.

The cells, maintained in culture from passages 2–8, grew adherent to plate and exhibited a typical spindle-shaped morphology as observed under inverted microscopy. No observable alterations in cell morphology were detected by optical microscopy. Following the examination of pluripotency markers (NANOG, Oct-4, and SOX2, data not shown), it became apparent that the cells preserved their pluripotent characteristics. An immunophenotypic evaluation using flow cytometry assay was performed to evaluate the expression of mesenchymal and hematopoietic markers. Results confirmed that the cells exhibited immunophenotypic characteristics consistent with those of a mesenchymal lineage when maintained in culture. Specifically, the cells were positive for the surface markers CD105, CD44, CD90 and CD73, while they were negative for CD14, CD34, CD19, CD45 and HLA-DR. The cellular differentiation capacity was also verified. In summary, our data confirm that the cells maintain their mesenchymal characteristics throughout the culture procedure and treatments (Dominici et al., 2006).

In relation to both environments to which the hPMSCs were exposed, growth kinetics assays revealed that hypoxic cultures exhibited a markedly higher cumulative population doubling potential. In addition to that our results strongly suggest that hypoxia improves the differentiation potential of these cells.

After thoroughly and rigorously defining and characterizing our hPMSCs, we tackled the experiments to investigate the potential of these cells in the process of adult CNS axotomized neurons axonal regeneration and restoration of neuronal activity. To address this goal, we used an *in vitro* co-culture model, previously established by Dr. Moreno-Flores`s team. Our group considered applying a similar approach to track and characterize the potential of hPMSCs in an axonal regeneration process. Specifically, hPMSCs and axotomized 2-month-old rat retinal ganglion cells (RGCs) shared the culture plate for 96 h and percentage of neurons with axon and mean axonal length per neuron were quantified (Portela-Lomba et al., 2020).

With our experimental approach, we achieved success getting 5 to 13-fold increase in the percentage of neurons with regenerated axons respect to RGCs in PLL. Moreover, the mean axonal length increased 4 and 24 times under normoxic and hypoxic conditions respectively. This result settled a very significant and promising result.

Numerous researchers have focused their efforts on exploring potential therapeutic strategies rooted in stem cell therapy for addressing conditions linked to neuronal degeneration or dysfunction. Nonetheless, while the predominant approach in these studies revolves around assessing the differentiation capacity of MSCs to support damaged cells, they ultimately conclude that the observed effects are mediated through paracrine signaling. A prevailing consensus among research collectives underscores the critical role of stem cells in facilitating the regeneration of injured nerves, primarily through the secretion of neurotrophic factors, including BDNF, NGF, NT-3 (Pastrana et al., 2007; Marconi et al., 2012; Taghi et al., 2012; Paradisi et al., 2014; Oses et al., 2017; Bari et al., 2018; Scheper et al., 2019; Trallori et al., 2019; Pisciotta et al., 2020; Saleh et al., 2020; Moonshi et al., 2022).

In line with previous studies, we confirmed that hPMSCs express the neurotrophic factors BDNF, NT3 and NGF. The Western blot results referred to the pro-factors expression indicate that pro-BDNF and pro NT-3 expression level were homogeneous along time in culture and with the different oxygen concentrations. However, the expression of the pro-NGF increased markedly when the cells were maintained in hypoxia.

Since the pro-factors should be cleaved intracellularly or extracellularly to generate their mature forms, we wanted to determine BDNF and NGF present in the conditioned media. It has been widely proven the role of BDNF as a key mediator in axonal regeneration by stimulating axonal growth and facilitating synaptic reorganization. It is demonstrated the role of BDNF in axonal regeneration of RGCs in co-culture with primary and immortalized rat OEG (Pastrana et al., 2007). In our system, we have found that hPMSCs (5 × 10^4^–10^5^) release BDNF in concentration between 25 ± 4.65 and 32 ± 2.66 pg/mL with no difference when we compared secretion by cells maintained in normoxia and hypoxia conditions.

NGF plays a crucial role in promoting the growth and survival of sensory neurons. Both the expression and the secretion of this factor appear to be hypoxia dependent. We have observed a twofold increase when comparing cultures maintained under hypoxia respect to normoxia.

It was of great interest to us to verify whether the RCGs regenerative effect of hPMSCs was due to a cell-to-cell contact, proximity-derived or a paracrine effect. To determine whether the previously tested effect was necessarily linked to a cell-cell contact we conducted indirect co-culture experiments, and secretome activity assays. The percentage of neurons exhibiting axonal regeneration in presence of conditioned media would quantify the contribution to this regeneration attributable to a neurotrophic impact of the hPMSCs. Furthermore, the mean axonal length per neuron provides an indicator for assessing the extent of axonal growth resulting from that neurotrophic influence. In addition to identified neurotrophic factors, other uncharacterized molecules secreted into the media may be playing a role in these regenerative parameters.

Significantly, in this experimental framework, we observed a reduction in the number of neurons exhibiting regenerated axons compared to the co-culture experiments involving RGCs and hPMSCs contact. Indeed, the number of regenerated RGCs closely mirrored the results obtained in RGC cultures without hPMSCs. Nevertheless, the paracrine impact of hPMSCs on axon length per neuron, under both normoxic and hypoxic conditions, exhibited an approximately three to fourfold increase when compared to the control RGCs cultured on PLL plates. These results suggest that hPMSCs play a key role as biological substrate strengthening the capacity of RGCs to regenerate their axons, partly through a paracrine mechanism. Additionally, we have shown that capacity to promote the highest axonal regeneration in this experimental paradigm depends on contact between the adult RGCs and the hPMSCs, as demonstrated for other regenerative cells, such as OEGs (Sonigra et al., 1999; Simón et al., 2011; Saleh et al., 2020).

We also wanted to confirm the regenerated RGCs functionality. Synaptic vesicles were localized distributed along the regenerated axons, suggesting their capacity to release neurotransmitters. In addition to that we assessed the regenerated axons’ ability to generate action potentials by characterizing the presence and activity of voltage-gated sodium channels (VGSCs). VGSCs play a pivotal role in the initiation and propagation of action potentials in neurons. Our findings demonstrate that the density of these channels is notably concentrated in the axon initial segment, a specialized region where action potentials are significantly greater than in the soma contributing to the distinctive electrical properties of this region. Based on our results, we can affirm that RGCs possess the molecular framework required for functionality.

Voltage-clamp experiments are a common method in neuroscience to measure the ionic currents in neurons. By controlling the membrane potential of the neuron, it is possible to measure the flow of ions in and out of the neuron during depolarization, providing valuable information about the neuron’s function and health (Sakmann and Neher, 1984).

In our experiments, we have confirmed the expression of VGSCs in the regenerated neurons. Moreover, we proceeded to compare the ionic currents generated by cells maintained in normoxia or hypoxia culture. Our findings demonstrate that regenerated neurons, under both environmental conditions, manifest transient sodium currents (I_Na_) and sustained potassium currents (I_K_) upon depolarization. Nevertheless, the amplitude of both sodium and potassium currents was notably reduced in the hypoxia-regenerated cells compared to those maintained under normoxic conditions, which could be due to the number and/or type of channels. In spite of these disparities, both cell types exhibited the capability to initiate action potentials, thereby affirming the electrophysiological activity of the regenerated cells representing without a doubt a biological breakthrough.

The study of the remarkably properties of hPMSCs, and the comprehensive understanding of all the molecules contributing to their ability to foster adult axonal regeneration undoubtedly warrant further investigation and approaches.

## Data Availability

The original contributions presented in the study are included in the article/Supplementary Material, further inquiries can be directed to the corresponding authors.
