# Supplementary material for: Human placenta-derived mesenchymal stem cells stimulate neuronal regeneration by promoting axon growth and restoring neuronal activity
Source: Front Cell Dev Biol. 2023 Dec 22;11:1328261. doi: 10.3389/fcell.2023.1328261 (PMC10766706; doi:10.3389/fcell.2023.1328261)
Supplement: Supplementary file 1 [file DataSheet2.pdf]

SUPPLEMENTARY INFORMATION

OIL RED-O STAINING

Adipogenesis differentiation was assessed using Oil-Red-O stain. Briefly, control and differentiated cultures were washed with phosphate-buffered saline (PBS 1X) and fixed with 10% formaline for 60 minutes. After that, formaline was removed and cultures were washed with distilled water and incubated in 60% isopropanol for 5 minutes at room temperature. Then, ~~Isopropanol~~ was removed and 2ml of Oil Red working solution were added to the cells for 10 minutes (stock solution: 300mg Oil Red (SIGMA) in 100 ml of 99% isopropanol; working solution: 6 ml of Oil Red O stock solution with 4 ml of distilled water). Cultures were rinsed with distilled water and let dry at room temperature. Intracellular lipid accumulation is stained as red droplets.

ALIZARIN RED STAINING

To demonstrate osteogenesis differentiation alizarin red stain was performed. Control and differentiated cultures were rinsed with phosphate-buffered saline (PBS 1X) and fixed with 10% formaline for 5 minutes. Then, cells were washed with distilled water twice and stained with alizarin red (SIGMA) (500 mg alizarin red in 25ml of distilled water) for 20 minutes. Cultures were rinsed with distilled water and let dry at room temperature. Calcified deposits are stained as red spots and were photographed by a microscope.

ALCIAN BLUE STAINING

After 14 days under differentiating conditions, media from cultures was removed. They were rinsed once with PBS, and cells were fixed with 4% paraformaldehyde (PFA) solution for 30 minutes. After fixation, cells were washed with PBS and stained with 1% alcian blue solution prepared in 0.1 N ~~HCL~~ for 30 minutes. ~~3.~~ Then, cells were rinsed three times with 0.1 N HCl. Acidity was neutralized washing the cells with distilled water. Blue staining indicates synthesis of proteoglycans by chondrocytes.

CD133 FLOW CYTOMETRY

hPMSCs grown in normoxia and hypoxia were assessed for CD133 expression and analyzed by flow cytometry. Briefly, cells were seeded at a concentration of 10^4 cells/cm^2 and maintained in culture until they reached 60%-70% confluence. Then cells were trypsinized, surface labeled with anti-CD133 (R&D Systems, Minneapolis, MN, USA, cat# MAB11331-SP) washed and then analyzed using a flow cytometer (Miltenyi Biotech). The data were analyzed with MACSQuantify™ Software.

E-CADHERIN IMMUNOSTAINING

hPMSCs cultures were fixed with 4% PFA to perform immunocytochemistry analysis. Samples were permeabilized in 0.5% Triton for 20 minutes and blocked in blocking solution (1% BSA) for 15 minutes at room temperature. Next, they were incubated with anti-E-cadherin (BD Transduction Laboratories, Franklin Lakes, NJ, USA, cat# MAB13881) in blocking solution at 1:50 dilution. Incubation was carried out at 37 °C for 1 hour. After washing with PBS 1X, the cells were incubated for another hour in a solution of PBS-TS containing Alexa Fluor 488 fluorescence-conjugated secondary antibody (1:500 dilution). HaCat cells were used as positive control.

VIMENTIN IMMUNOSTAINING

MSC cultures were fixed with 4% PFA. Then, they were permeabilized and blocked in blocking solution (1X PBS, 0.1% triton, 1% FBS) for 30 minutes at room temperature. Next, they were incubated with anti-vimentin antibody (Abcam, Cambridge, UK, cat# ab24525, 1:1000) in blocking solution at 4 °C overnight. After washing with PBS 1X, the cells were incubated for one hour in a solution of PBS-TS containing Alexa 647 fluorescence-conjugated secondary antibody (1:500 dilution).
