# Supplementary figures and images for: Human placenta-derived mesenchymal stem cells stimulate neuronal regeneration by promoting axon growth and restoring neuronal activity
Source: Front Cell Dev Biol. 2023 Dec 22;11:1328261. doi: 10.3389/fcell.2023.1328261 (PMC10766706; doi:10.3389/fcell.2023.1328261)

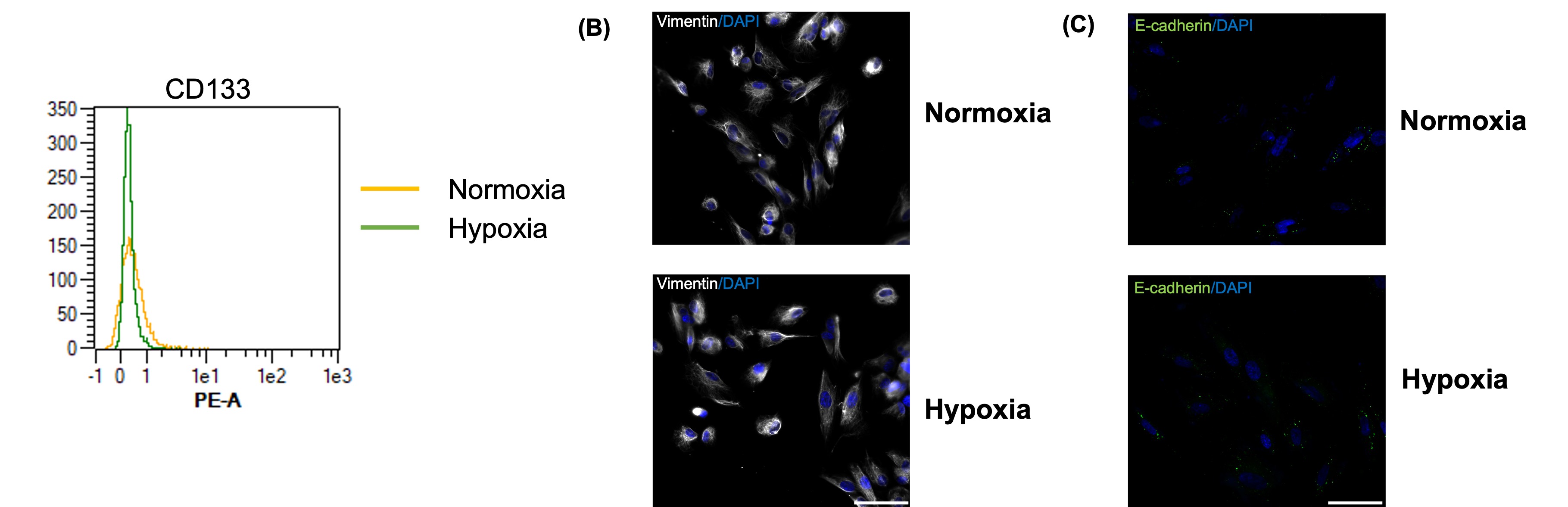

Supplement: Supplementary file 2 [file Image1.JPEG]
