# Supplementary material for: Human placenta-derived mesenchymal stem cells stimulate neuronal regeneration by promoting axon growth and restoring neuronal activity
Source: Front Cell Dev Biol. 2023 Dec 22;11:1328261. doi: 10.3389/fcell.2023.1328261 (PMC10766706; doi:10.3389/fcell.2023.1328261)
Supplement: Supplementary file 3 [file DataSheet1.PDF]

CAPTION SUPPLEMENTARY FIGURE 1

Supplementary figure 1. Immunostaining of hPMSCs. (A) Flow cytometry shows hPMSCs negative expression of CD133. (B) hPMSCs cultures cells were fixed and immunostained with antibody against vimentin (type III intermediate filament protein, white) and nuclear staining with DAPI (blue). Scale bar: 100μm. (C) Representative immunofluorescence of E-cadherin (epithelial cadherin, green) and nuclear staining with DAPI (blue). Scale bar: 100μm.
